# The Glasgow Prognostic Score and stricture site can predict prognosis after endoscopic duodenal stent placement for malignant gastric outlet obstruction

**DOI:** 10.1038/s41598-022-13209-x

**Published:** 2022-06-13

**Authors:** Yu Takamatsu, Nao Fujimori, Tsukasa Miyagahara, Yuta Suehiro, Toyoma Kaku, Ken Kawabe, Akihisa Ohno, Kazuhide Matsumoto, Masatoshi Murakami, Katsuhito Teramatsu, Ayumu Takeno, Takamasa Oono, Yoshihiro Ogawa

**Affiliations:** 1 Department of Medicine and Bioregulatory Science, Graduate School of Medical Sciences, Kyushu University, 3-1-1 Maidashi, Higashi-ku, Fukuoka, 812-8582 Japan; 2 Department of Gastroenterology, Nakatsu Municipal Hospital, Nakatsu, Japan; 3 Department of Gastroenterology, Clinical Research Institute, National Hospital Organization Kyushu Medical Center, Fukuoka, Japan

**Keywords:** Gastrointestinal diseases, Cancer

## Abstract

Endoscopic duodenal stent (DS) placement for malignant gastric outlet obstruction (GOO) is rapidly increasing in clinical practice; however, the most suitable patient candidates for DS placement have not been determined. One hundred and thirty-five patients with GOO who underwent DS placement in three Japanese referral centers between January 2010 and October 2019 were retrospectively evaluated. Overall survival (OS) after DS placement, technical/clinical success rates, adverse events, and predictive factors affecting OS after DS placement were also analyzed. The median OS after DS placement of all patients was 81 (7–901) days. Technical and clinical success rates were 99.3% and 83.7%, respectively. The GOO Scoring System score significantly increased before and after DS placement (0.9 vs. 2.7, *P* < 0.001). The procedure-related complication rate was 6.0%. All 19 patients (14.1%) with stent occlusion underwent endoscopic re-intervention successfully. Multivariate analyses revealed chemotherapy after DS placement (*P* = 0.01), stricture site in D3 (distal part of the papilla) (*P* = 0.01), and a Glasgow Prognostic Score (GPS) of 0–1 before duodenal stent placement (*P* < 0.001) were factors significantly associated with prolonged OS. In conclusion, patients with a GPS of 0–1 and D3 stricture who are tolerant of chemotherapy are suitable candidates for DS placement.

## Introduction

Gastric outlet obstruction (GOO) occurs as a result of a narrowing in the region of the gastroduodenum, which induces failed or delayed passage of gastric contents from the stomach to the jejunum^1^. GOO is a common complication of advanced pancreato-biliary cancer, distal gastric cancer, duodenal cancer, and metastases from other malignancies^2^. Once malignant GOO occurs, patients experience episodes of abdominal fullness, nausea, vomiting, and the difficulty of adequate oral intake^3^. These symptoms significantly impact performance status and quality of life, resulting in difficulties with cancer treatment and shortening of patient survival^4^. GOO is usually treated with surgical gastrojejunostomy (GJ) or insertion of an endoscopic duodenal stent (DS). Although it remains unknown whether DS is superior to GJ for malignant GOO, DS placement is rapidly increasing in clinical practice since it is a less-invasive treatment that promptly relieves symptoms^2,5,6^. However, the long-term efficacy and safety of DS have not been fully elucidated in the era of advancing oncological treatments, and several issues, such as short overall survival (OS) after DS placement, high rate of re-intervention, and multiple type of cancers as the cause of GOO, remain unsolved.

Recently, simple parameters of systemic inflammation, such as the neutrophil-to-lymphocyte ratio (NLR) and the Glasgow Prognostic Score (GPS), have been recognized as prognostic markers for patients with various cancers including pancreato-biliary malignancies^7–10^. However, the relationship between these systemic inflammatory markers and the prognosis of patients with DS placement is scarcely known.

Treatment after DS placement is another important issue because it significantly affects OS in patients with DS. Several previous reports revealed the efficacy of chemotherapy after DS placement^3,11,12^, but some patients, following DS placement, are unable to receive chemotherapy due to low performance status in daily clinical practice. Thus, it has not been determined who will benefit most from DS placement despite the widespread application of DSs.

In this study, we evaluated the safety and efficacy of endoscopic DS placement for patients with GOO in three referral centers located in the Kyushu region of Japan. We aimed to evaluate the clinical effectiveness of DS placement and to identify prognostic factors associated with survival after DS placement.

## Methods

### Study population and data collection

This retrospective study was conducted at three referral centers to evaluate the efficacy and long-term outcome of patients with GOO who underwent endoscopic insertion of a DS. Patients with GOO were recruited from each hospital between January 2010 and October 2019. The patients with malignant GOO who underwent DS placement during the above period were included in this study. The exclusion criteria were as follows: poor general condition (high risk of endoscopic procedure), resectable diseases, and surgically altered anatomy except for Billroth I reconstruction. We reviewed the following clinical data: age, sex, symptoms, Eastern Cooperative Oncology Group Performance Status (PS), NLR and GPS before DS placement, Gastric Outlet Obstruction Scoring System (GOOSS) score, primary cancer type, site of stenosis, procedures for duodenal stenosis and biliary obstruction, re-intervention, OS, adverse events, such as procedure-related complications and stent dysfunction, and oncological treatments. The primary endpoint was OS after DS placement. The secondary endpoints were technical/clinical success rates, rate of adverse events, predictive factors for clinical success, and predictive factors affecting OS after DS placement. We obtained informed consent for the DS placements from all patients. This study was conducted in accordance with Helsinki Declaration, and the study protocol was approved by the Ethics Committee of Kyushu University (approval number: 2020-75), Nakatsu Municipal Hospital (approval number: NMH2019044), and Kyushu Medical Center (approval number: 20C136).

### Endoscopic procedures

Endoscopic DS placements were performed under conscious sedation induced by an intravenous injection of midazolam and/or pentazocine. A lateral-viewing duodenal endoscope (TJF-260V, Olympus Medical Systems, Tokyo, Japan) or a forward-viewing gastrointestinal endoscope (CF-H260AI, Olympus Medical Systems, Tokyo, Japan) was used according to the site of the stricture. First, an endoscope was inserted at the proximal site of stenosis with a catheter, and a guidewire passed through the stricture. After confirming the site and length of duodenal stenosis with a contrast medium, the DS was deployed across the stricture under endoscopic and fluoroscopic guidance. According to the site and length of a stricture, a WallFlex DS (6, 9, or 12 cm in length, 22 mm in body diameter; Boston Scientific, Marlborough, MA, United States), Niti-S DS (uncovered type 6, 8, 10, or 12 cm in length, covered type 8 or 10 cm in length, 22 mm in body diameter; Taewoong Medical, Seoul, Korea) or Evolution DS (6, 9, or 12 cm in length, 22 mm in body diameter; Cook Medical, Winston-Salem, NC, United States) was selected based on the endoscopist’s preference.

### Definitions

The extent of oral intake was evaluated using the GOOSS: 0, no oral intake; (1) exclusively liquid diet; (2) exclusively soft solid diet; and (3) low-residue or full diet^2^. We recorded the GOOSS within the 7 days before and after placement of the DS, and the best score was applied to the analysis.

The sites of the duodenal obstruction were defined as D1, D2, and D3, as previously reported; D1 and D3 strictures were in the proximal and distal parts of the papilla, respectively, and a D2 stricture was defined as having involvement in the papilla^13^.

OS was defined as the duration from the date of DS placement to the date of death. Technical success was defined as the adequate endoscopic placement of the DS across the stenosis. Clinical success was defined as the achievement of a GOOSS score of 3 after DS placement.

### Statistical analyses

Statistical analyses were performed using the JMP version 15 software (SAS Institute Inc., Cary, NC, United States). GOOSS scores before and after DS placement were compared using a Wilcoxon signed-rank test. Predictive factors for clinical success were analyzed using logistic regression models. OS was analyzed using the Kaplan–Meier method. Univariate and multivariate analyses were conducted using the Cox proportional hazard model to reveal the predictive factors for OS. The level of significance was set at *P* < 0.05.

## Results

### Clinical characteristics of enrolled patients

A total of 138 patients with GOO who underwent endoscopic DS placement were enrolled in the study. Three patients were excluded, two of whom with surgically altered anatomy had a stent insertion for another object, such as resolution of obstructive jaundice and afferent loop syndrome. One patient had surgical treatment after DS placement. Thus, 135 patients were analyzed, and their clinical characteristics are summarized in Table 1.

**Table 1:** Characteristics of enrolled patients.

**Sex**	
Male	80 (59.3)
Female	55 (40.7)
Age (median [range])	72.0 (42–95)
**Performance status at DS placement**	
0	20 (14.8)
1	48 (35.6)
2	46 (34.1)
3	21 (15.6)
NLR (median)	4.6
**GPS**	
0	20 (15)
1	45 (33)
2	70 (52)
**Cancer type**	
PDAC	92 (68.1)
Cholangiocarcinoma	18 (13.3)
Gall bladder cancer	8 (5.9)
Gastric cancer	4 (3.0)
Ampullary cancer	3 (2.2)
Duodenal cancer	2 (1.5)
Hepatocellular carcinoma	2 (1.5)
Colon cancer	2 (1.5)
Renal cell carcinoma	2 (1.5)
Neuroendocrine carcinoma	1 (0.7)
Breast cancer	1 (0.7)
**Disease status**	
Locally advanced disease	37 (27.4)
Metastatic disease	98 (72.6)
**Site of stricture**	
D1	64 (47.4)
D2	36 (26.7)
D3	35 (25.9)
**Type of DS**	
Niti-S^a^	59 (43.7)
WallFlex^b^	51 (37.8)
Evolution^c^	25 (18.5)
**Presence of biliary stricture**	91 (67.4)
Before duodenal stenosis	59 (43.7)
Simultaneously with duodenal stenosis	24 (17.8)
After duodenal stenosis	8 (5.9)
Oncological treatment before DS placement	79 (58.5)
Chemotherapy after DS placement	48 (35.6)

Categorical data are presented as number (percent), continuous data as median (range).

*DS* duodenal stent, *GPS* Glasgow Prognostic Score, *NLR* neutrophil-to-lymphocyte ratio, *PDAC* pancreatic ductal adenocarcinoma.

^a^Taewoong Medical, Seoul, Korea.

^b^Boston Scientific, Marlborough, MA, United States.

^c^Cook Medical, Winston-Salem, NC, United States.

Of the 135 patients, 67 (49.7%) had poor PS (2 or 3), and 65 (48.1%) had a GPS of 0 or 1. The most common primary cancer was pancreatic ductal adenocarcinoma (PDAC) (68.1%), followed by cholangiocarcinoma (13.3%). Ninety-eight patients (72.6%) had metastatic diseases. The site of GOO was most often located in D1 (47.4%), and the numbers of D2 and D3 strictures were comparable. Regarding the type of DS, Niti-S, WallFlex, and Evolution, which were all uncovered type, were used in 59, 51, and 25 patients, respectively (Supplementary Table 1). Ninety-one patients (67.4%) had biliary strictures, and most of them occurred before or simultaneously with GOO. After DS placement, approximately one-third of the patients received chemotherapy.

### Clinical outcomes of DS placement

The clinical outcomes of DS placement are shown in Table 2. The rate of technical success was 99.3%. One patient had technical failure due to stent migration immediately after stent placement. This patient was treated successfully with re-positioning of DS endoscopically on the next day. The median duration from DS placement to oral intake was 2 days. The mean GOOSS scores before and after DS placement were 0.9 ± 0.093 and 2.7 ± 0.057, respectively (Table 2). Therefore, the mean GOOSS score significantly improved after DS placement (*P* < 0.001). Of the 135 patients, 113 achieved a GOOSS score of 3 after DS placement, indicating the rate of clinical success was 83.7%. The type of DS did not affect the clinical outcomes.

**Table 2:** Clinical outcomes of duodenal stent placement.

Technical success (n, %)	134 (99.3)	
Clinical success (n, %)	113 (83.7)	
GOOSS before DS placement	0.92 ± 0.09	
GOOSS after DS placement	2.73 ± 0.01	*P* < 0.001

### Adverse events of DS placement and endoscopic re-intervention

The adverse events of DS placement and details of endoscopic re-intervention are shown in Table 3. Procedure-related complications, including cholangitis, perforation, and aspiration pneumoniae, were observed in four, one, and one patient, respectively. Jejunal perforation occurred 16 days after DS placement due to stent migration. Stent migration developed in two of 135 patients (1.5%).

**Table 3:** Adverse events of duodenal stent placement.

Adverse events (n, %)	26 (19.3)
**Procedure-related complications (n, %)**	6 (4.0)
Cholangitis	4 (3.0)
Perforation*	1 (0.7)
Aspiration pneumoniae	1 (0.7)
**Stent dysfunction (n, %)**	
Stent migration^a^	2 (1.5)
Stent occlusion	19 (14)

^a^One case is overlapping.

Stent occlusion due to tumor ingrowth or overgrowth occurred in 19 of 135 patients (14.1%) during the observation period. Stent patency of 19 patients with stent occlusion was 66 (5–418) days, and all patients successfully underwent endoscopic re-intervention with additional DS placement. Details of patients with stent occlusion and endoscopic re-intervention are shown in Table 4.

**Table 4:** Characteristics of 19 patients with duodenal stent occlusion.

Sex, male	14 (73.4)
Age (median [range])	69 (45–86)
**Performance status at DS placement**	
0	5 (26.3)
1	6 (31.6)
2	4 (21.1)
3	4 (21.1)
NLR (median)	4.7
**GPS**	
0	1 (5.3)
1	12 (63.2)
2	6 (31.6)
**Cancer type**	
PDAC	11 (57.9)
Non-PDAC	8 (42.1)
**Disease status**	
Locally advanced	7 (36.8)
Metastatic disease	12 (63.2)
**Site of stricture**	
D1	10 (52.6)
D2	5 (26.3)
D3	4 (21.1)
**Type of DS**	
Niti-S	6 (31.6)
WallFlex	8 (42.1)
Evolution	5 (26.3)
**Presence of biliary stricture**	
Before duodenal stenosis	8 (42.1)
Simultaneously with duodenal stenosis	2 (10.5)
After duodenal stenosis	3 (15.8)
Oncological treatment before DS placement	14 (73.7)
Chemotherapy after DS placement	11 (57.9)
Stent patency (days, median [range])	66 (5–418)
**Re-intervention using an additional stent**	
Niti-S	13 (68.4)
WallFlex	4 (21.1)
Evolution	2 (10.5)

Categorical data are presented as number (percent), continuous data as median (range).

*DS* duodenal stent, *GPS* Glasgow Prognostic Score, *NLR* neutrophil-to-lymphocyte ratio, *PDAC* pancreatic ductal adenocarcinoma.

### Predictive factors for improvement of QOL after DS placement

To reveal the predictive factors for improved QOL, such as an achievement of GOOSS 3 after DS placement, we compared the clinical characteristics between the patients with clinical success (GOOSS 3 after DS placement) and failure (GOOSS 0–2 after DS placement) (Supplementary Table 2). On multivariate analyses, PS 0–1 and oncological treatment before DS placement were significantly associated with GOOSS 3 (improved QOL) after DS placement (Table 5).

**Table 5:** Predictive factors for achievement of GOOSS 3 after DS placement.

Factor	Univariate analysis			Multivariate analysis		
n = 135	OR	95% CI	*P*-value	OR	95% CI	*P*-value
Sex, male	1.25	0.50–3.16	0.62			
Age < 72	1.1	0.44–2.75	0.84			
PS 0–1	5.88	1.87–18.5	0.002	5.42	1.60–18.3	0.007
NLR < 4.6 before DS placement	1.98	0.77–5.09	0.16			
GPS 0–1 before DS placement	2.91	1.06–7.99	0.04	1.89	0.62–5.74	0.26
PDAC	1.61	0.63–4.12	0.32			
Locally advanced disease	2.61	0.72–9.43	0.14			
Site of stricture, D3	2.5	0.69–9.04	0.16			
Presence of biliary stricture	1.22	0.47–3.17	0.68			
Oncological treatment before DS placement	3.76	1.42–9.98	0.008	4.34	1.54–12.2	0.006

*CI* confidence interval, *DS* duodenal stent, *GOOSS* Gastric Outlet Obstruction Scoring System, *GPS* Glasgow Prognostic Score, *HR* hazard ratio, *NLR* neutrophil-to-lymphocyte ratio, *PDAC* pancreatic ductal adenocarcinoma, *PS* performance status.

### Predictive factors for OS after DS placement

**Figure 1:**
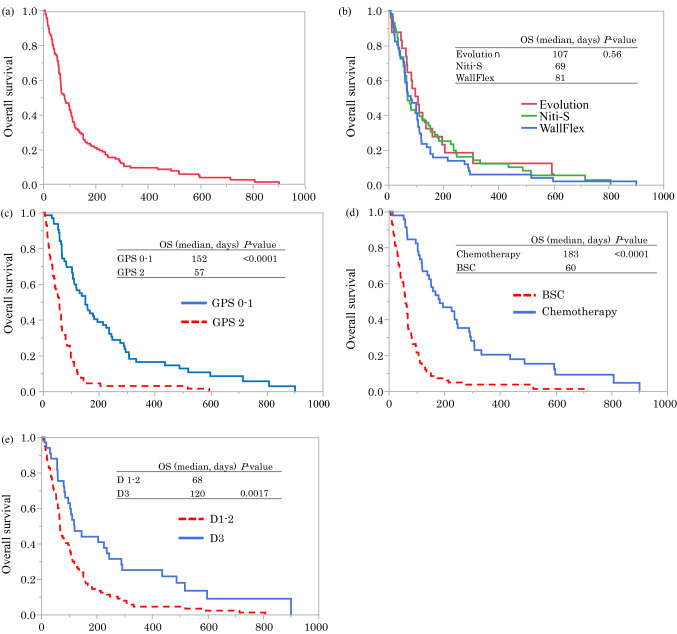
Kaplan–Meier curves for overall survival (OS) after duodenal stent (DS) placement. (**a**) OS of all patients enrolled in this study. The median OS after DS placement was 81 (7–901) days. (**b**) OS according to stent types. No significant differences were observed. (**c**) OS according to a GPS of 0–1 or 2. The median OS for a patient with a GPS of 0–1 was significantly longer compared with that for a patient with a GPS of 2. (**d**) OS of patients with chemotherapy or best supportive care (BSC) after DS placement. The median OS of the chemotherapy group was significantly longer compared with that of the BSC group. (**e**) OS of patients with duodenal strictures in D1-2 or D3. The median OS of the D3 stricture group was significantly longer compared with that of D1-2 stricture group.

The median OS of all patients enrolled was 81 (range 7–901) days (Fig. 1a). The type of DS did not lead to significant differences in OS after stent placement (Fig. 1b). Based on the univariate analysis, a PS 0–1, GPS 0–1 before DS placement, stricture site in D3, GOOSS score of 3 after DS placement, and chemotherapy after DS placement were significantly favorable for OS. The results of multivariate analyses showed that GPS 0–1 before DS placement (hazard ratio [HR] 0.43; 95% confidence interval [CI] 0.28–0.66, *P* < 0.001), locally advanced disease (HR 0.57; 95% CI 0.37–0.88, *P* = 0.01), chemotherapy after DS placement (HR 0.55; 95% CI: 0.34–0.88, *P* = 0.01), and stricture site in D3 (HR 0.55; 95% CI 0.35–0.87, *P* = 0.01) were factors significantly associated with prolonged OS (Table 6).

**Table 6:** Univariate and multivariate analyses for overall survival after DS placement.

Factor	Univariate analysis			Multivariate analysis		
n = 135	HR	95% CI	*P*-value	HR	95% CI	*P*-value
Sex, male	0.72	0.51–1.04	0.08			
Age < 72	1.06	0.74–1.50	0.76			
PS 0–1	0.57	0.40–0.81	0.002	0.81	0.53–1.23	0.33
NLR < 4.6 before DS placement	0.41	0.28–0.59	< 0.0001	0.71	0.47–1.05	0.09
GPS 0–1 before DS placement	0.30	0.20–0.45	< 0.0001	0.43	0.28–0.66	0.0001
PDAC	0.86	0.59–1.25	0.43			
Locally advanced disease	0.42	0.27–0.63	< 0.0001	0.57	0.37–0.88	0.01
Site of stricture, D3	0.51	0.33–0.78	0.002	0.55	0.35–0.87	0.01
Presence of biliary stricture	1.32	0.89–1.95	0.17			
GOOSS 3 after DS placement	0.46	0.29–0.74	0.001	0.9	0.53–1.52	0.69
Oncological treatment before DS placement	0.82	0.57–1.18	0.29			
Chemotherapy after DS placement	0.29	0.20–0.44	< 0.0001	0.55	0.34–0.88	0.01

*CI* confidence interval, *DS* duodenal stent, *GOOSS* Gastric Outlet Obstruction Scoring System, *GPS* Glasgow Prognostic Score, *HR* hazard ratio, *NLR* neutrophil-to-lymphocyte ratio, *PDAC* pancreatic ductal adenocarcinoma, *PS* performance status.

Figure 1c–e show the Kaplan–Meier curves of OS according to the GPS before DS placement, chemotherapy after DS placement, and site of stricture, respectively. The median OS for patients with a GPS of 0–1 was significantly longer than the patients with a GPS of 2 (152 vs. 57 days, *P* < 0.0001). OS in the chemotherapy group was significantly longer compared to the best supportive care group (183 vs. 60 days, p < 0.0001). In addition, OS of the patients with a D3 stricture was significantly longer than those with a D1 or D2 stricture (120 vs. 68 days, *P* = 0.002).

## Discussion

This study demonstrated the efficacy and safety of DS placement for GOO, which resulted from pancreato-biliary and other malignancies in clinical settings in the Kyushu region of Japan. The technical and clinical success rates were high, and complications were acceptable; however, patient prognosis after DS placement was very poor, a median of 81 days. We found that a GPS of 0–1, D3 stricture, or receipt of chemotherapy after DS placement led to longer OS.

PDAC is widely recognized as a highly lethal disease. Two retrospective studies from Japan reported the median survival times after DS placement for patients with advanced pancreatic cancer of 89.5^14^ and 95^15^ days, which were comparable to that of the results of the present study. The high proportion of patients with PDAC (over 60%) might have been the reason for poor prognosis in this study. However, OS after DS placement did not significantly differ between patients with and without PDAC (85 vs. 81 days, *P* = 0.43) in our cohort. Similarly, Miyabe et al.^16^ reported that survival durations after DS placement in patients with pancreato-biliary and other cancers were comparable. In a larger study by Oh et al.^3^ (292 patients), post-stent placement survival was similar between patients with pancreatic and non-pancreatic cancer (2.7 vs. 2.4 months). A recent study from a French cancer center, including patients with various cancer types, also revealed a poor prognosis of four months after DS placement^17^. Although endoscopic DS placement itself is a safe procedure and tends to be the first-line treatment for malignant GOO, endoscopists should recognize that GOO is still a poor prognostic marker for patients with cancers, regardless of origin, in the era of advancement of oncological treatment.

Given that chemotherapy was selected for tolerable patients, it was reasonable that chemotherapy after DS placement was the most favorable factor for prolonged OS. Similarly, several studies indicated chemotherapy after DS placement as a predictive factor for longer OS^3,16^. Oh et al.^3^ reported that chemotherapy post-stent placement was significantly associated with better post-stent placement survival in both patients with pancreatic and non-pancreatic cancer. Clinicians should consider the induction or re-induction of chemotherapy in tolerable patients after DS placement to achieve longer OS.

A systemic inflammatory marker, GPS, was also a prognostic factor after DS placement in our study. Although there are few reports regarding systemic inflammation in patients who underwent DS placement, Kobayashi et al.^12^ and Sugiura et al.^18^ reported that an NLR ≥ 5 or NLR ≥ 4 was significantly associated with poor prognosis in patients with pancreatic cancer. Although an NLR < 4.6 in our study was not significant and the optimal cut-off value of the NLR remains unknown, the NLR may be a prognostic factor for patients with DS placement. A GPS or modified GPS was reported to be a predictor for patients with stenting in other fields, such as esophageal stent^19^, biliary obstruction^20,21^, and obstructive colorectal cancer^22^; however, the role of GPS has not been clarified in patients with DS placement. To the best of our knowledge, we are the first to reveal the importance of a GPS before DS placement in patients with GOO. We should take systemic inflammatory markers, such as the GPS, into consideration when deciding on a DS placement because it is a very simple and easily assessable marker.

**Figure 2:**
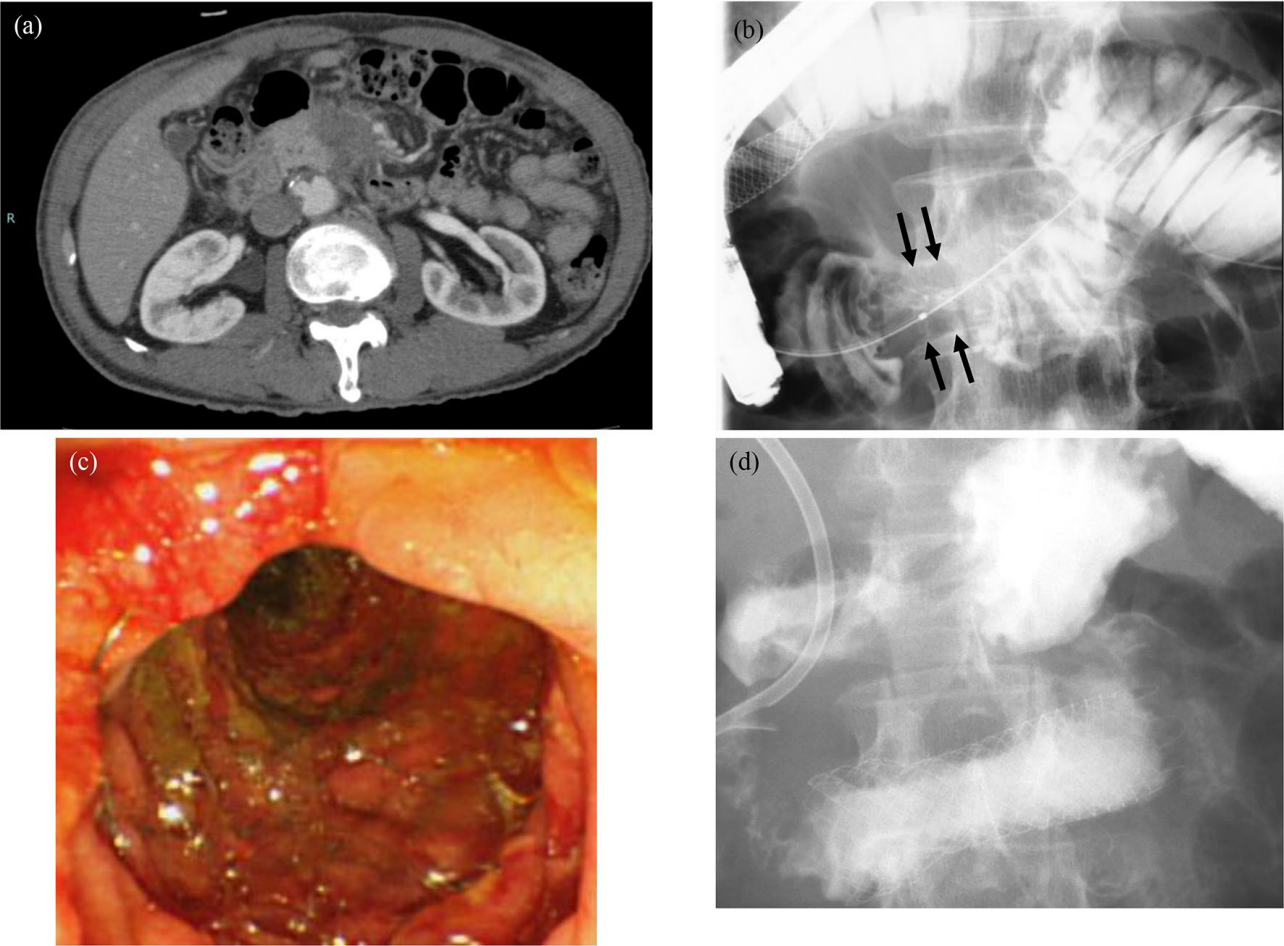
Images of a 70-year-old man with metastatic pancreatic cancer. Both his Gastric Outlet Obstruction Scoring System (GOOSS) and Glasgow Prognostic Score were 0 before duodenal stent (DS) placement. (**a**) Computed tomography revealed a pancreatic tumor with duodenal invasion. (**b**) Fluoroscopic imaging shows a severe D3 stricture (arrows). Endoscopic (**c**) and fluoroscopic (**d**) images show the resolution of the stricture after DS placement, which has improved the GOOSS to 3. He received chemotherapy and achieved long-term survival (901 days) without re-intervention of the DS.

The stricture site D3 was another significant favorable factor for prolonged OS after DS placement, which was the new finding in the present study. For patients with malignant GOO, the stricture site was divided into three or four portions, D1, D2, D3, and D4, in the previous studies^14,15,17,23^. However, it has not been clarified whether prognosis depends on the stricture site. Several studies demonstrated that the stricture site was not a significant prognostic factor for duration of survival or stent patency^15,16,23–25^. Jung et al.^25^ reported that the clinical success rate was significantly greater in patients with stenosis of the peri-pyloric region (pylorus and duodenal bulb) than those of the duodenal region (second and third portions of the duodenum), although the stent patency period was not different. Theoretically, patients with a D3 stricture have less involvement of the biliary tract, which may facilitate prompt induction of chemotherapy after DS placement. These patients might be suitable candidates for DS placement. Clinical images of a representative patient who received chemotherapy after DS placement for a D3 stricture are shown in Fig. 2.

In this study, we performed uncovered DS placement as an initial procedure in all patients with GOO. In a recent randomized prospective study regarding malignant GOO from Japan^26^, the rate of stent migration after uncovered DS placement was as low as 2.2% (4/184), which was similar with our results. We think that stent migration rarely occurs if expert endoscopists carefully perform DS placement using an uncovered stent. On the other hand, the weak point of uncovered DS is frequent stent occlusion due to the tumor ingrowth or overgrowth. Actually, 19 of 135 patients (14.1%) had stent occlusion in our study and required additional DS placement. We should take into consideration the more frequent occlusion of DS when prolonged OS is achieved due to advances of oncological treatment in the future.

This study has several limitations. First, we retrospectively analyzed patients with malignant GOO who had been treated with an endoscopic DS placement. A comparative analysis of DS placement to surgical bypass was not performed in this study. A comparative analysis in future studies could give insight into the factors that make DS the more suitable or better option. Second, this study included heterogenous primary cancers, not just pancreatic cancers, and various sites of malignancies. Third, chemotherapy regimen for pancreatic cancer or biliary cancer was not consistent due to a long-term study period, which may affect clinical outcomes including OS. Although a larger study is required to validate the benefit of DS for GOO, our results have important implications regarding clinical decision-making for patients with malignant GOO.

In conclusion, we demonstrated the safety and efficacy of endoscopic DS placement for patients with GOO in real clinical settings. Patients with a GPS of 0–1 and a D3 stricture who are tolerant of chemotherapy might be suitable candidates for endoscopic DS placement.

## Supplementary Information

Supplementary Tables.
