# Supplementary material for: The Glasgow Prognostic Score and stricture site can predict prognosis after endoscopic duodenal stent placement for malignant gastric outlet obstruction
Source: Sci Rep. 2022 Jun 13;12:9746. doi: 10.1038/s41598-022-13209-x (PMC9192609; doi:10.1038/s41598-022-13209-x)
Supplement: Supplementary file 1 — Supplementary Tables. [file 41598_2022_13209_MOESM1_ESM.docx]

Supplementary Table 1. Clinical characteristics according to stent types

|  | WallFlex (n=51) | Niti-S (n=59) | Evolution (n=25) |
| --- | --- | --- | --- |
| Sex, male | 28 (54.9) | 35 (59.3) | 17 (68.0) |
| Age (median [range]) | 71 (49-94) | 72 (42-95) | 77 (60-93) |
| Performance status at DS placement |  |  |  |
| 0 | 3 (5.9) | 12 (20.3) | 5 (20.0) |
| 1 | 17 (33.3) | 21 (35.6) | 10 (40.0) |
| 2 | 20 (39.2) | 20 (33.9) | 6 (24.0) |
| 3 | 11 (21.6) | 6 (10.2) | 4 (16.0) |
| NLR (median) | 4.7 | 4.67 | 4.1 |
| GPS |  |  |  |
| 0 | 7 (13.7) | 9 (15.3) | 4 (16.0) |
| 1 | 18 (35.3) | 19 (32.2) | 8 (32.0) |
| 2 | 26 (51.0) | 31 (52.5) | 13 (52.0) |
| Cancer type |  |  |  |
| PDAC | 38 (74.5) | 40 (67.8) | 14 (56.0) |
| Non-PDAC | 13 (25.5) | 19 (32.2) | 11 (44.0) |
| Site of stricture |  |  |  |
| D1 | 28 (54.9) | 26 (44.1) | 10 (40.0) |
| D2 | 10 (19.6) | 19 (32.2) | 7 (28.0) |
| D3 | 13 (25.5) | 14 (23.7) | 8 (32.0) |
| Presence of biliary stricture |  |  |  |
| Before duodenal stenosis | 24 (47.1) | 25 (42.4) | 10 (40.0) |
| Simultaneously with duodenal stenosis | 15 (29.4) | 4 (6.8) | 5 (20.0) |
| After duodenal stenosis | 5 (9.8) | 2 (3.4) | 1 (4.0) |
| Oncological treatment before DS placement | 35 (68.6) | 31(52.5) | 13 (52.0) |
| Technical success (n, %) | 50 (98.0) | 59 (100) | 25 (100) |
| Clinical success (n, %) | 44 (86.3) | 50 (84.7) | 19 (76.0) |
| Procedure-related complication (n, %) | 4 (7.8) | 2 (3.4) | 0 (0.0) |
| Stent dysfunction (n, %) | 8 (15.7) | 6 (10.2) | 5 (20.0) |
| Categorical data are presented as number (percent), continuous data as median (range). DS, duodenal stent; GPS, Glasgow Prognostic Score; NLR, neutrophil-to-lymphocyte ratio; PDAC, pancreatic ductal adenocarcinoma | | | |

Supplementary Table 2. Clinical characteristics between GOOSS 3 and GOOSS 0-2 after DS placement

|  | GOOSS 3 (n=113) | GOOSS 0-2 (n=22) | P-value |
| --- | --- | --- | --- |
| Sex, male | 68 (60.2) | 12 (54.5) | 0.62 |
| Age (median [range]) | 72 (42-95) | 73.5 (60-91) | 0.56 |
| Performance status at DS placement |  |  | <0.01 |
| 0 | 20 (17.7) | 0 (0) |  |
| 1 | 44 (38.9) | 4 (18.2) |  |
| 2 | 34 (30.1) | 12 (54.5) |  |
| 3 | 15 (13.3) | 6 (27.3) |  |
| NLR (median) | 4.51 | 5.46 | 0.16 |
| GPS |  |  | 0.03 |
| 0-1 | 59 (52.2) | 6 (27.3) |  |
| 2 | 54 (47.8) | 16 (72.7) |  |
| Cancer type |  |  | 0.33 |
| PDAC | 79 (69.9) | 13 (59.1) |  |
| Non-PDAC | 34 (30.1) | 9 (40.9) |  |
| Disease status |  |  | 0.11 |
| Locally advanced disease | 33 (29.2) | 3 (13.6) |  |
| Metastatic disease | 80 (70.8) | 19 (86.4) |  |
| Site of stricture |  |  | 0.19 |
| D1 | 50 (44.2) | 14 (63.6) |  |
| D2 | 31 (27.4) | 5 (22.7) |  |
| D3 | 32 (28.3) | 3 (13.6) |  |
| Presence of biliary stricture |  |  | 0.66 |
| Before duodenal stenosis | 48 (42.5) | 11 (50.0) |  |
| Simultaneously with duodenal stenosis | 23 (20.4) | 1 (4.5) |  |
| After duodenal stenosis | 6 (5.3) | 2 (9.1) |  |
| Oncological treatment before DS placement | 72 (63.7) | 7 (31.8) | 0.01 |
| Categorical data are presented as number (percent), continuous data as median (range). DS, duodenal stent; GPS, Glasgow Prognostic Score; NLR, neutrophil-to-lymphocyte ratio; PDAC, pancreatic ductal adenocarcinoma | | | |
